# Decomposition rate and property changes of deadwood across an altitudinal gradient: a case study in the Babia Góra Massif, Poland

**DOI:** 10.1038/s41598-025-14019-7

**Published:** 2025-08-01

**Authors:** Adam Górski, Ewa Błońska, Jarosław Lasota

**Affiliations:** Department of Ecology and Silviculture, Faculty of Forestry, University of Agriculture in Krakow, 29 Listopada 46 Str., 31-425 Kraków, Poland

**Keywords:** Vertical zonation, Decomposition process, Forest ecosystems, Water repellency, Woody debris, Forestry, Carbon cycle, Element cycles, Boreal ecology, Forest ecology, Ecology

## Abstract

The decomposition of deadwood is a key process in the biogeochemical cycle of forests, affecting water retention, soil structure and biodiversity. The aim of this study is to understand how the rate of deadwood decomposition changes depending on the location in the altitude gradient in mountain forest ecosystems. Additionally, the study investigates how the physical properties of wood vary with elevation. The experiment was conducted on the slopes of the Babia Góra Massif, where wood samples of four species (beech, fir, spruce, maple) were placed at three altitudes (800, 1000 and 1200 m above sea level). After 30 months, laboratory analyses were carried out on the density, porosity, mass loss and hydrophobicity of wood. In the case of coniferous wood (spruce and fir), the decomposition process proceeded at a similar rate across all altitudes, but more slowly compared to deciduous species. In contrast, hardwood decomposed more rapidly at lower altitudes, likely due to higher temperatures, greater microbial activity, and soil conditions more favorable to hardwood-decaying organisms. Wood decomposition led to a decrease in density and an increase in porosity, and hydrophobicity increased with altitude. The study provides new data on the dynamics of wood decomposition in the context of changing thermal and moisture conditions. The results can be used in conservation and management strategies for mountain forests.

## Introduction

Deadwood is a fundamental element of forest ecosystems^1^. It plays a key role in the cycling of elements such as carbon, nitrogen and phosphorus, constituting a long-term store of nutrients that slowly return to the soil during its decomposition^2^. It is also an important reservoir of organic carbon, which has a direct impact on climatic balance^3^. In addition, deadwood is an important habitat for many organisms, such as fungi, plants, invertebrates, birds and small mammals, which use as shelter, site for breeding, source of food or is associated with saproxylic organisms^4^. Its presence contributes to the maintenance of biodiversity, providing ecological niches for specialist species^5^. Finally, deadwood supports forest health by influencing water retention, soil structure and tree regeneration^6^. Deadwood supports forest health by providing habitat and recycling nutrients, but in mountain coniferous forests, excessive deadwood can also promote bark beetle outbreaks, posing a threat to forest vitality.

The decomposition of deadwood is a complex process that depends on the interactions of biotic and abiotic factors^7^. The most important abiotic factors include temperature, which affects the activity of enzymes of wood-decomposing microorganisms, and humidity, which determines the availability of water for metabolic processes^8^. Biotic factors include the diversity and activity of microorganisms (bacteria and fungi), which are the main agents of decomposition, and saproxylic insects that aid in wood fragmentation^9^. The rate of decomposition also depends on the characteristics of the wood itself, such as hardness, chemical composition (proportion of lignin, cellulose, hemicelluloses), and the content of minerals and water^10^. These changes affect the physical properties of wood, which gradually becomes less compact and more susceptible to microorganism colonization^11^.

Wood decomposition leads to changes in its properties, including alterations in the wood’s structure. Water repellency (WR) is recognized as a crucial property that aids in understanding water balance^12^. The water drop penetration time (WDPT) test, which measures the time required for a single water droplet to infiltrate, is the most commonly used method for estimating WR^13^. WR is a crucial parameter in understanding water absorption and storage dynamics within forest ecosystems. Water repellency (WR) and water drop penetration time (WDPT) are key indicators of the hydrophilic or hydrophobic nature of deadwood, directly linked to the stages of wood decomposition^14^.

The elevation gradient is a unique tool for studying environmental changes in a short geographical space^11^. With increasing altitude, temperature decreases, air humidity amplitude increases, and the growing season becomes shorter^15^. These differences have a significant impact on biological processes, including the rate of deadwood decomposition^16^. Previous studies have focused mainly on the influence of factors such as humidity, temperature, or tree species on deadwood decomposition in lowland, homogeneous^17^. However, relatively little attention has been paid to studying wood decomposition in the context of altitude gradients. In particular, there is a lack of knowledge about how these gradients affect changes in the physical and chemical properties of wood during decomposition.

The aim of this study is to understand how the rate of deadwood decomposition changes depending on the location in the altitude gradient in mountain forest ecosystems. Additionally, the study investigates how the physical properties of wood vary with elevation. Specifically, the study focuses on: (1) comparing the rate of wood decomposition across an altitude gradient; (2) demonstrating the changes in physical properties and water repellency of deadwood, depending on the decomposition stage. Altitude gradients can serve as “natural laboratories” to test predictions under real-world conditions. The altitude gradient not only provides unique insight into ecosystem responses to climate change, but also enables precise design of adaptation and conservation strategies in mountainous regions worldwide.

## Materials and methods

### Study site

**Fig. 1:**
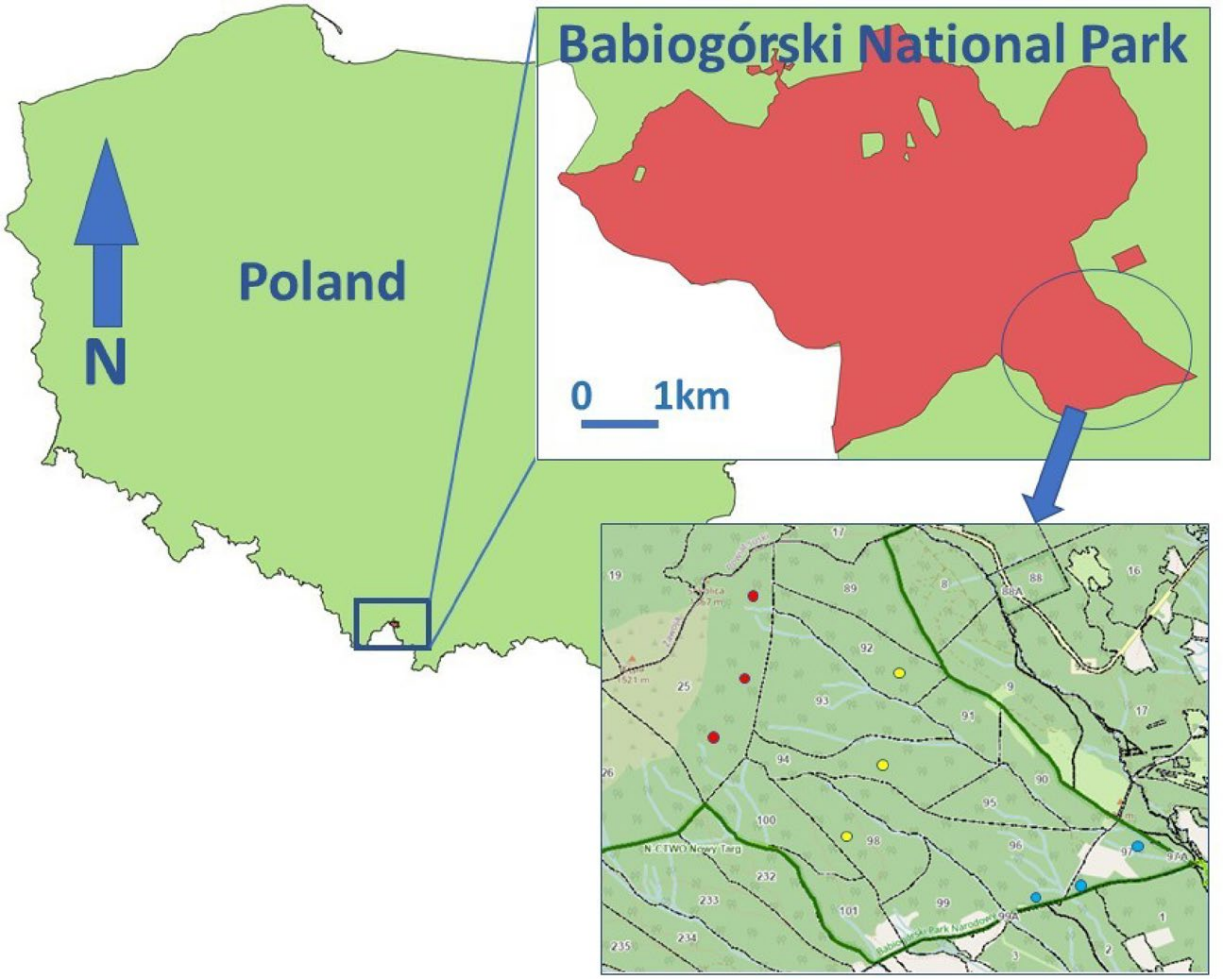
Location of study plots in altitude gradient (red points—1200 m a.s.l.; yellow points—1000 m a.s.l.; blue points—800 m a.s.l.)

**Fig. 2:**
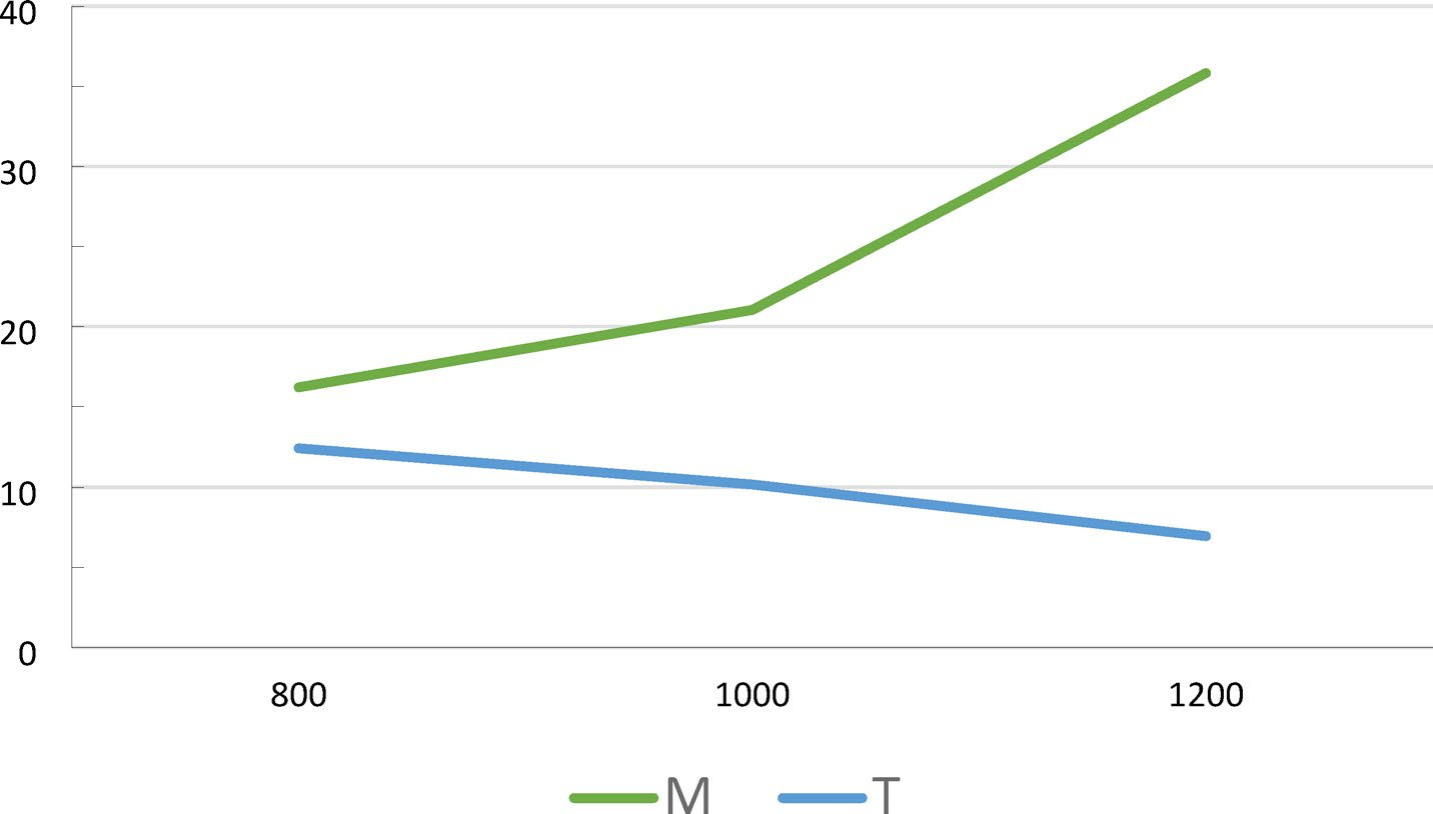
Moisture (M—%) and temperature (T—^o^C) of the surface horizon of soil in altitude gradient (800, 1000 and 1200 m above sea level).

The research was conducted on the southern slopes of the Babia Góra Massif (49°35’18’’N; 19°32’23’’E). Three transects were established along an altitudinal gradient. Within each transect, research plots were positioned at elevations of 800 m a.s.l., 1000 m a.s.l., and 1200 m a.s.l (Fig. 1). The transects were located in areas with Dystric Cambisols characterized by a silty loam texture. At an altitude of 800 m above sea level there was a mixed stand of fir, spruce and beech, at an altitude of 1000 m a.s.l. there was a spruce stand with an admixture of beech and fir, and at an altitude of 1200 m a.s.l. there was a spruce stand. The plots were dominated by forest stands with moderate canopy density, which was consistent across all research areas. Canopy density was determined using a four-level scale reflecting the degree to which the space is filled by the tree stand, as proposed in the Forest Management Instruction (2012). Study plots were located on south-facing slopes with an inclination of 10 degrees. The research sites were situated in mature forest areas that have not been previously used for agricultural purposes and have historically remained forested. During plot selection, sites affected by erosion, landslides, and colluvial processes were deliberately excluded. Average annual temperatures recorded for the study plots at 800 m a.s.l. it was 5.1 °C, at 1000 m a.s.l. it was 4.0 °C and at 1200 m a.s.l. it was 3.1 °C (Fig. 2).

In March 2021 a mesocosm experiment was set up on each plot. The mesocosms, consisting of a 20-cm-high collar in the form of a square measuring 1 m by 1 m, were placed directly on the soil surface. Mesocosms were placed at each of three elevations and altitude gradients. Three transects were included in the study. Four types of wood were included in the experiment––common beech (*Fagus sylvatica* L.), silver fir (*A. alba* Mill.), Norway spruce (*Picea abies* (L.) H.Karst) and sycamore maple (*Acer pseudoplatanus* L.). Wood of the four species, in form of cubes in five repetitions was placed in each mesocosm. The cubes were 50 × 50 × 22 mm. All samples of wood were dried before starting the experiment in the field. The wood was dried at 105 °C for 48 h in a laboratory dryer. The samples for the experiment were prepared from one piece of wood (large branch) to maintain their comparability. The wood samples were placed directly on the soil surface in each mesocosm and after 30 months they were subjected to laboratory analyses. The experiment ended in September 2023. Temperature and humidity sensors were installed in each mesocosm. After completing the field experiment, the wood samples were analyzed in the laboratory.

### Wood analysis

Wood analyzes in the laboratory began with determining the volume. Wood samples were measured using a caliper. Then we measured the fresh weight of the wood samples. The samples were dried at 105 °C for 48 h and weighed after drying. The following characteristics were calculated:

$${\text{Dd}}\,=\,{\text{Wd/V }}[{\text{g}}\;{\text{c}}{{\text{m}}^{\text{3}}}]$$

*where* Dd = dry density, Wd = dry weight, and V = volume of fresh sample,

$${\text{MR }}={\text{ }}\left( {{\text{Dd/Dfw}}} \right)\cdot{\text{1}}00{\text{ }}\left[ \% \right]$$

*where* MR = rest mass, Dd = dry density, and Dfw = density of fresh wood,

$${\text{MD}}\,=\,{\text{1}}00--{\text{MR }}\left[ \% \right]$$

*where* MD = mass decline, MR = rest,

$${\text{Por }}={\text{ }}([{\text{Dws}}--{\text{Dd}}]/{\text{Dws}})\cdot{\text{1}}00{\text{ }}\left[ \% \right]$$

*where* Por = porosity, Dws = density of wood substance, and Dd = dry density.

The above properties were determined after the experiment was completed. The density of fresh wood (Dfw) was determined prior to commencing the experiment. The wood substance’s density (Dws) was established in accordance with Krzysik^18^.

Water repellency (WR) was assessed by measuring water drop penetration time (WDPT) following Wessel’s method. The method has been tested in the context of decaying wood in previous studies^14^. In this test, 5 drops of distilled water (approximately 0.05 ml each) were placed on the wood surface using a laboratory micropipette, with three replicates for each sample. The time (in seconds) required for water infiltration was recorded and compared to standard values according to the classification by Täumer et al.^19^. This classification defines seven levels of repellency, from “wettable” (WDPT less than 5 s) to “extremely water repellent” (WDPT exceeding 6 h) (see Table 1). The experiment was conducted in laboratory conditions at a stable air temperature of 21 ± 1 °C, with water droplets at the same temperature. WDPT analysis included two moisture conditions off wood: natural and dry.

**Table 1:** Classes of WDPT used in this study.

Classes	Class 0	Class 1	Class 2	Class 3	Class 4	Class 5	Class 6
WDPT	< 5 s	5–60 s	1–10 min	10–60 min	1–3 h	3–6 h	> 6 h
	wettable	slightly water repellent	strongly water repellent	severely water repellent	extremely water repellent	extremely water repellent	extremely water repellent

### Statistical analysis

The properties of wood depending on species and position in the altitude gradient are presented on boxplots. Generalized linear mixed-effects model (GLM) was used to determine the role of species type and altitude and the interaction between them on the wood characteristics. Principal Component Analysis (PCA) was used to explore the relationships between the examined characteristics. Additionally, PCA was applied to classify species based on the wood properties examined. Statistical analyses were performed using the programming language R in R Studio.

## Results

**Fig. 3:**
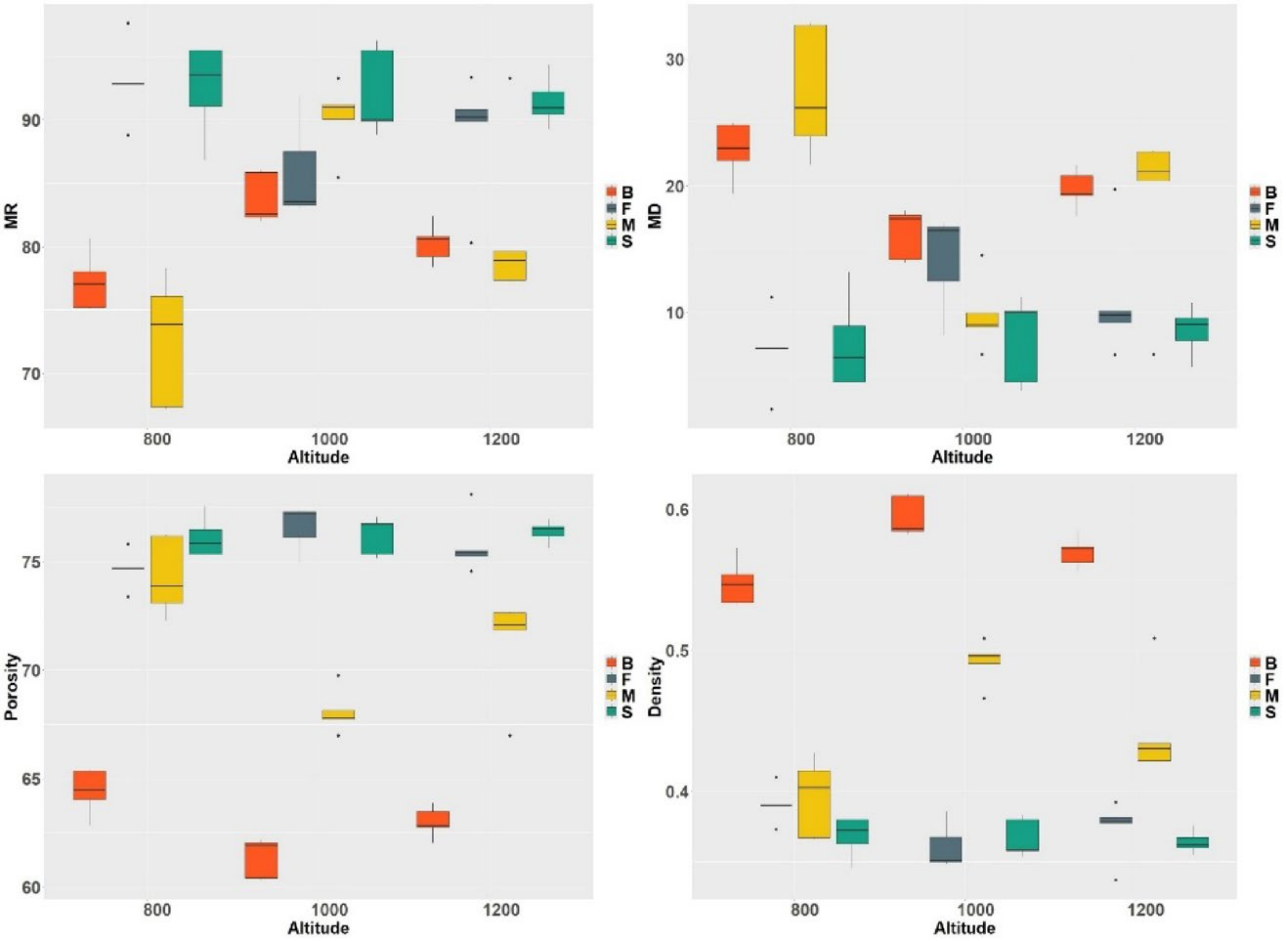
Physical properties of wood of different species depending on the position in the altitude gradient (above sea level) (B: beech, F: fir, M: maple, S: spruce; dry density of deadwood (g.cm^− 3^), MR: mass rest (%), MD: mass decline (%), Por: porosity (%)).

Soil moisture increases with altitude, with the highest values observed at 1200 m a.s.l. For instance, the average soil moisture at 1200 m was approximately 30%, while at 800 m it was around 20% (Fig. 3). On the other hand, soil temperature showed an opposite trend: the highest values were recorded at the lowest altitude, while at 1200 m a.s.l. it dropped.

The analysis of physical properties of deadwood indicates significant relationships between the wood species and altitude (Fig. 3). Mass rest (MR) was the highest for spruce and fir at altitudes of 1000 and 1200 m a.s.l., reaching values of approximately 90–91% and 83–90%, respectively. Deciduous species, such as beech and maple, showed lower moisture content – for beech at 800 m a.s.l., MR was about 77%, while for maple it was approximately 74%. Mass decline (MD) was the greatest at the lowest altitude (800 m a.s.l.), particularly for maple and beech, where the values reached approximately 32% and 23%, respectively. At altitudes of 1000 and 1200 m a.s.l., MD for fir was around 17% and 10%, respectively, while for spruce it was approximately 10% and 8%, respectively. Deciduous species at these altitudes exhibited lower mass decline. Wood porosity (Por) increased with altitude, especially for spruce and fir, reaching values of around 75% at 1000 and 1200 m a.s.l. Beech and maple showed lower porosity, at approximately 62–64% across all altitudes. The dry density of deadwood was the highest for beech at 1000 m a.s.l. – around 0.58 g·cm⁻³ – and for beech at 1200 m a.s.l. – approximately 0.57 g·cm⁻³. The lowest density was recorded for fir and spruce at 1000 and 1200 m a.s.l., where values dropped to around 0.35–0.37 g·cm⁻³. Maple at 800 m a.s.l. showed a density of approximately 0.40 g·cm⁻³ (Fig. 3).

**Fig. 4:**
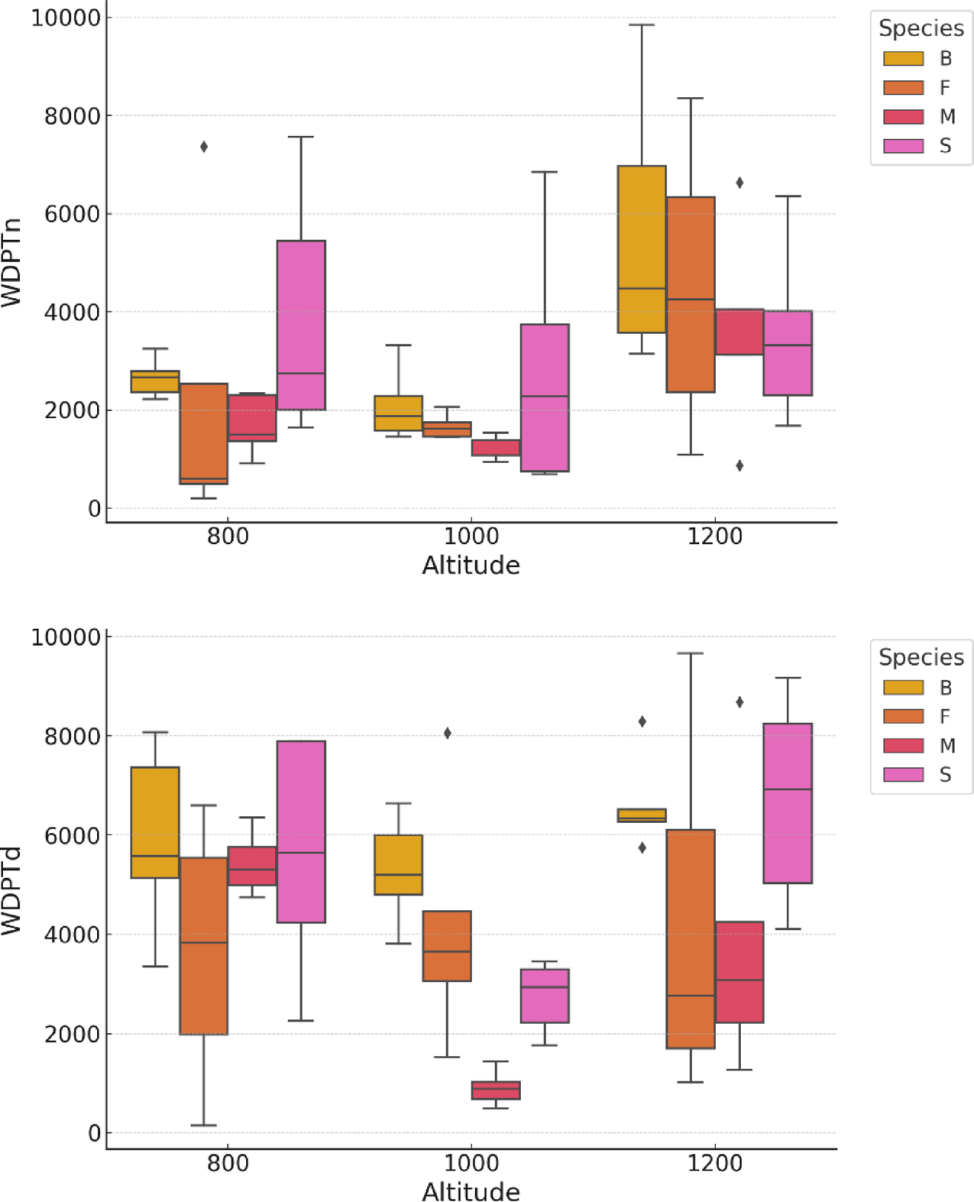
Water drop penetration time (s) of different species depending on the position in the altitude gradient (above sea level) (B: beech, F: fir, M: maple, S: spruce; WDPTn: Water Drop Penetration Time in natural moisture (sec), WDPTd: Water Dropp Penetration Time in dray deadwood (sec)).

Figure 4 shows the variability in WDPT depending on the wood species and position in the altitude gradient. For naturally moist wood, WDPT increases with altitude across all studied species. Spruce wood exhibited reduced water penetration even at lower altitudes, such as 800 m a.s.l. For dry wood, greater species variability in WDPT was observed, particularly at 1000 and 1200 m a.s.l.

The results of a GLM statistical analysis assessing the effects of wood species, altitude, and their interaction on selected physical properties of wood are presented in Table 2. Wood species had a significant effect on all analyzed traits (*p* < 0.001). Porosity and density were particularly dependent on species, with the highest F-values of 132.59 and 126.78. The Altitude plays a significant role in shaping the physical properties of wood. Additionally, altitude gradient significantly influenced WDPTn (F = 7.34, *p* < 0.001) and WDPTd (F = 6.19, *p* < 0.001). The interaction of species and altitude had a significant effect on all physical properties of wood (*p* < 0.001), indicating complex relationships between specific species characteristics and the altitude gradient.

**Table 2:** GLM analysis results for Deadwood characteristics.

	MR		MD		Porosity		Density		WDPTn		WDPTd	
	F	*p*	F	*p*	F	*p*	F	*p*	F	*p*	F	*p*
Species	34.9	**0.00**	34.9	**0.00**	373.5	**0.00**	373.5	**0.00**	1.44	0.24	4.35	**0.00**
Altitude	6.1	**0.00**	6.2	**0.00**	10.8	**0.00**	10.8	**0.00**	7.34	**0.00**	6.19	**0.00**
Species * altitude	9.9	**0.00**	9.8	**0.00**	10.7	**0.00**	10.8	**0.00**	0.74	0.61	1.85	0.10

**Fig. 5:**
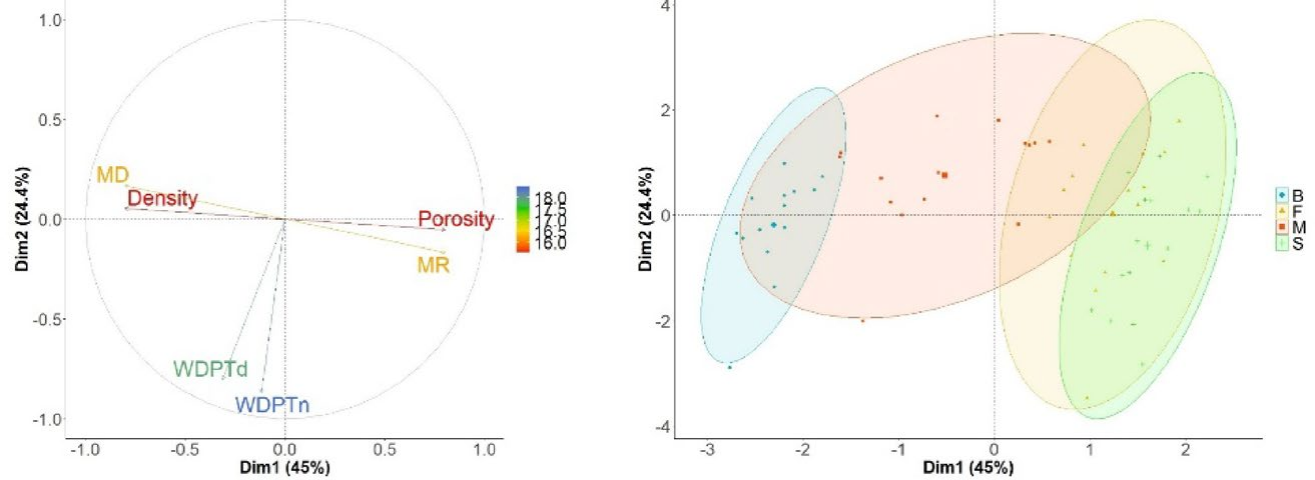
Projection of variables on the plane of the first and second PCA factors (B: beech, F: fir, M: maple, S: spruce; MR: mass rest, MD: mass decline, WDPTn: Water Drop Penetration Time in natural moisture, WDPTd: Water Dropp Penetration Time in dray deadwood).

The Principal Component Analysis (PCA), in which the data has been reduced to two principal components (Principal Component 1 and Principal Component 2), is shown in Fig. 5. Factors 1 and 2 explain over 69% of the variability in the studied characteristics. Factor 1 is related to the physical properties of the tested wood, while factor 2 is related to the WDPT. The figure shows the distinction between deciduous and coniferous species in terms of wood properties.

## Discussion

In the conducted experiment, we wanted to check how the decomposition process of the same wood samples placed in mesocosms in the altitude gradient is shaped. It could be expected that the thermal and moisture conditions changing with increasing altitude would significantly affect the decomposition process of the wood used in the experiment. There are reports in the previous studies on the importance of thermal and moisture factors on wood decomposition. It is believed that in temperate climate conditions, an increase in temperature stimulates the decomposition processes of deadwood^20^while humidity is also emphasized as an important factor influencing the decomposition process^8^. Low humidity associated with periods of water deficit and drought may be the reason for limiting the rate of decomposition^21^although Stienen et al.^22^ showed that hyphae of basidiomycetes can moisten dry wood, as long as there is a source of moisture nearby, and thus change the conditions in which wood decomposition occurs. Stability of moisture content is important for efficient wood decomposition, but too high wood moisture content limiting oxygen processes can also lead to slower decomposition processes^17^. In the conditions of the experiment, there was a significant decrease in temperature in the altitude gradient associated with the location above sea level. Humidity was stable and increased with the elevation above sea level, but its level certainly did not limit the availability of oxygen necessary for the decomposition process. As for the obtained results, with the elevation gradient, we found a slightly different trend in the progress of decomposition associated with different species. In the case of coniferous wood (spruce and fir), the decomposition process proceeded at a similar rate across all altitudes, but more slowly compared to deciduous species. Can we conclude that the increase in humidity turned out to be a key factor in this case and more important than the deterioration of thermal conditions, as was found, for example, in the Mediterranean climate zone^23^? In our opinion, precipitation and temperature—both closely linked to altitude—should be considered the primary factors influencing decomposition processes along the elevation gradient. However, additional factors, such as soil properties (e.g. pH), may also contribute. For instance, acidic soils present across all elevations can support microbiota adapted to such conditions, which are capable of decomposing coniferous wood. We are aware that a more complete explanation of the rate of wood decomposition would be provided by analysis of the microorganisms involved in this process.

In the Babia Góra National Park, coniferous species are present across the entire altitudinal gradient, contributing to soil acidification and leaching throughout. In such conditions, microbiota develops, adapted to the acidic environment, having the ability to decompose coniferous wood species that naturally dominate in this environment^24^ Species such as beech and sycamore naturally occur and co-create communities in lower altitudes (< 1000 m). In locations of 800 m, which are the natural environment of development of these species, we also found the fastest decomposition of wood of these species. This may indicate that in the conditions in which these species naturally exist, there is an appropriate composition of microorganisms in the soil environment that decompose detritus from these trees, and in our experience, influencing the correspondingly faster decomposition of the wood samples used. Therefore, while altitude correlates strongly with temperature and precipitation, it is likely that a combination of climatic and edaphic (soil-related) factors together shaped the observed decomposition patterns. The importance of the composition of microbiota and mezofauna on the course and rate of deadwood decomposition processes has been proven in a number of studies^23,25–28^. The wood of deciduous species has a different chemical composition (different proportion of cellulose to lignin, higher share of additional wood-building substances, especially chemicelluoses, phenols, or different chemical composition of lignin), which force a certain specialization of decomposers performing the decomposition process^29^. The wood of the analyzed deciduous species contains a greater number of parenchyma cells, which show higher resistance to degradation by fungi causing brown rot, intensively decomposing the xylem of coniferous species^30^. An interesting finding of our experiment is that deciduous wood decomposed by white rot fungi, which degrade lignin and extractives, showed reduced water absorbability. Some studies also draw attention to the influence of wood variability of one species related to the density of annual rings, the share of late wood, the size of conducting vessels or the presence or absence of heartwood^25,31^ and the influence of these features on decomposition processes. Being aware of these phenomena, we used wood samples of each species prepared from the same type of wood (from one larger, homogeneous piece) to avoid the influence of differences in the internal structure of the wood on the experimental results.

The observed changes in physical properties in decaying wood are a consequence of the advancement of decomposition processes. The loss of wood mass is directly related to the degree of decomposition and is the inverse of the value of the remaining wood mass. With the advancement of the decomposition process, the bulk density of wood decreases, which has already been confirmed many times in previous studies^17,32^. The measurement of WDPT on decaying wood gave an interesting result. The longest water absorption time was found for wood that decomposed at the highest position (1200 m) and this tendency appeared for wood of three species - fir, beech and maple. Such a result would not be surprising if it were not for the lack of a clear correlation of WDPT with wood porosity and MD at the same time. Beech and maple wood at 1200 m altitude showed a relatively low degree of decomposition, in this case a high WDPT value seems logical, while in the case of fir at the highest locations a high MD and high porosity were found, while water absorption was slowed down. It is difficult to clearly justify the obtained result. A probable cause may be an increase in the concentration of substances in the decaying wood that cause higher hydrophobicity of the wood tissue. Such substances may be terpenes and terpenoids as well as wax and resin substances^33^. Based on the conducted research, it is difficult to clearly determine the mechanism of the processes that occurred. Further research is necessary to confirm the above assumptions.

## Conclusions

This study demonstrates that deadwood decomposition along an altitudinal gradient is not governed by a single climatic factor, but rather by a complex interplay of temperature, moisture, soil properties, and species-specific traits. While higher humidity and lower temperatures at elevated sites would traditionally be expected to slow decomposition, we found that coniferous wood (spruce and fir) decomposed at a similar rate across all altitudes, likely due to the presence of specialized microbial communities adapted to acidic soils in these zones. In contrast, hardwood species (beech and sycamore) decomposed fastest at lower elevations, where they naturally occur and where microbial decomposer communities are well-adapted to their chemical structure. Our findings highlight that decomposition dynamics are strongly species-dependent and that both environmental conditions and the functional characteristics of decomposer organisms must be considered when predicting decomposition rates across landscapes. Furthermore, the observed changes in wood water repellency (WDPT) with altitude suggest that decomposition may influence not only nutrient cycling but also hydrological properties of forest soils. Although detailed microbiota analyses were not conducted in this study, it would be worthwhile to include such data in future research to better elucidate the mechanisms underlying differences in wood decomposition rates depending on local conditions and substrate type.

## Data Availability

The data is supplied in the manuscript. However, in the event of a request for raw data, it will be issued upon request.Contact person: Adam Górski.
